# Insights into a Protein-Nanoparticle System by Paramagnetic Perturbation NMR Spectroscopy

**DOI:** 10.3390/molecules25215187

**Published:** 2020-11-07

**Authors:** Yamanappa Hunashal, Cristina Cantarutti, Sofia Giorgetti, Loredana Marchese, Federico Fogolari, Gennaro Esposito

**Affiliations:** 1 Science Division, New York University Abu Dhabi, 129188 Abu Dhabi, UAE; yh45@nyu.edu; 2 Dipartimento di Area Medica, Università di Udine, P.le Kolbe, 4, 33100 Udine, Italy; 3 Institute de Chimie, UMR CNRS 7272, Université Côte d’Azur, Université de Nice Sophia Antipolis, Parc Valrose, 06108 Nice, CEDEX 2, France; cristina.cantarutti@gmail.com; 4 Dipartimento Medicina Molecolare, Università di Pavia, Via Taramelli 3, 27100 Pavia, Italy; sofia.giorgetti@unipv.it (S.G.); loredana.marchese@unipv.it (L.M.); 5 Dipartimento di Matematica, Informatica e Fisica, Università di Udine, Viale delle Scienze, 33100 Udine, Italy; federico.fogolari@uniud.it; 6 Instituto Nazionale Biostrutture e Biosistemi, Viale Medaglie d’Oro 305, 00136 Roma, Italy

**Keywords:** protein-nanoparticle interactions, protein NMR, amyloidogenic proteins, nitroxide paramagnetic perturbation, spin label extrinsic probes, Tempol, β2-microglobulin

## Abstract

Background: The interaction between proteins and nanoparticles is a very relevant subject because of the potential applications in medicine and material science in general. Further interest derives from the amyloidogenic character of the considered protein, β2-microglobulin (β2m), which may be regarded as a paradigmatic system for possible therapeutic strategies. Previous evidence showed in fact that gold nanoparticles (AuNPs) are able to inhibit β2m fibril formation in vitro. Methods: NMR (Nuclear Magnetic Resonance) and ESR (Electron Spin Resonance) spectroscopy are employed to characterize the paramagnetic perturbation of the extrinsic nitroxide probe Tempol on β2m in the absence and presence of AuNPs to determine the surface accessibility properties and the occurrence of chemical or conformational exchange, based on measurements conducted under magnetization equilibrium and non-equilibrium conditions. Results: The nitroxide perturbation analysis successfully identifies the protein regions where protein-protein or protein-AuNPs interactions hinder accessibility or/and establish exchange contacts. These information give interesting clues to recognize the fibrillation interface of β2m and hypothesize a mechanism for AuNPs fibrillogenesis inhibition. Conclusions: The presented approach can be advantageously applied to the characterization of the interface in protein-protein and protein-nanoparticles interactions.

## 1. Introduction

The interaction of proteins with nanoparticle (NP) systems is a very challenging issue that has many implications in physical chemistry as well as in biomedical and biochemical applications [1]. Depending on the NP charge, size, shape, and chemical functions on the surface, proteins may be adsorbed onto that surface, concentrated in a layer named corona, or experience labile interactions and exchange with bulk solution. There are several experimental strategies that can be employed to assess the interaction between NP and proteins, ranging from direct inspection by microscopy or suitable spectroscopic techniques, e.g., UV, fluorescence, surface-enhanced Raman spectroscopy, etc., to the indirect inference based on the assay of the protein function through the related biological or cellular response. More detailed information can be obtained also by other techniques such as NMR, although the application viability is restricted to those systems where the protein exchange can be exploited to gain information on the transiently bound states that are unobservable by NMR because of the large sizes. However, magnetic resonance can be employed over a wider scale range if electron resonance is considered, provided the systems under consideration respond to free paramagnetic species or can host suitable paramagnetic probes on the proteins or the NPs. For NMR applications, the most convenient NP size window to modulate the protein interaction is the medium-size range, i.e., 5–20 nm, where the effects of the NP shape, charge, and surface chemistry can be tuned for the scopes of interest.

Over the last few years, we have carried out systematic investigations on the interaction of amyloidogenic protein models and citrate-coated or alkanethiolate-coated gold NPs (AuNPs) with diameters of 3.6, 5, and 7.5 nm [2,3,4,5,6,7]. We worked in particular on β2-microglobulin (β2m) and variants thereof that represent a paradigmatic example of amyloidogenic protein misfolding [8,9]. β2m naturally occurs in class I major histocompatibility complex on the surface of antigen presenting cells, in conjunction with a larger domain. Due to renal failure and consequent high concentration from impaired clearance [8], or because of a mutation [9], a pathologic fibrillar aggregation of β2m takes place, leading to amyloid deposition in patients undergoing long-term hemodialysis or aged individuals with genetically inherited mutation.

NP interaction studies were conducted on wild-type β2m [10,11,12], the naturally occurring amyloidogenic mutant D76N β2m [9,13] and ∆N6 β2m, which is a variant devoid of the first six residues that is found only in natural fibrils of the wild-type species [14]. Contrary to the expectations based on earlier results [15], the protein solutions with AuNPs were stable for several months, and no evidence of increased aggregation or partial unfolding was observed. The occurrence of uneven patterns of signal attenuation was indicative of a preferential interface of fast exchange with AuNPs [2,3,4]. With D76N β2m, the most amyloidogenic variant of β2m that fibrillates by agitation at neutral pH, the presence of citrate-stabilized AuNPs inhibited fibrillogenesis by interfering with the early aggregation steps of the protein that are crucial for the protofibril nucleus formation, as inferred from NMR, QCMD (Quartz Crystal Microbalance with Dissipation monitoring), and MD (Molecular Dynamics) [3,4,6].

Recently, we have revived the use of water-soluble nitroxides such as Tempol to explore the exchange dynamics of β2m [16]. Tempol and similar stable free radicals had been formerly employed as extrinsic probes for identifying the protein exposed locations, based on the paramagnetic perturbation of the NMR signals induced by the unpaired electron of the radical over accessible molecular surfaces [17,18,19,20]. The same paramagnetic perturbation measured under non-equilibrium conditions of the NMR magnetization determines an attenuation pattern that differs from the corresponding profile obtained under equilibrium conditions, i.e., with fully relaxed NMR magnetization. While in the latter conditions, the extent of NMR attenuation reflects the proximity to the unpaired electron and therefore the accessibility of or the distance from the molecular surface, the attenuation retrieved under non-equilibrium conditions of magnetization recovery can map also the locations of hindered accessibility or μs-to-ms exchange events, by identifying slower or faster relaxing nuclei, respectively, with respect to the average relaxation rate enhancement brought about by the nitroxide probe [16].

Here, we show the application of this novel use of Tempol attenuation to gain insights into the interactions that wild-type β2m establishes with citrate-coated AuNPs. The analysis of the ternary system protein/AuNP/spin-label probe is conducted with respect to all the NMR- (Nuclear Magnetic Resonance) and ESR- (Electron Spin Resonance) accessible controls involving only two components of the system, which are based also on the previously reported evidence [2,3,4,5,6,7,16].

## 2. Results

Using extrinsic spin labels such as nitroxides to extract structural information requires testing the reliability of their non-specific probe behavior [17,18,19,20]. ESR spectra of Tempol in the absence and presence of β2m had previously shown that only statistical encounters occur between the free radical and the protein, as inferred from the invariance of linewidths and amplitudes of the superimposed spectra [16]. For the ternary system protein + AuNPs + Tempol, the ESR trace superposition for the three controls (Tempol, Tempol + AuNPs, and Tempol + β2m) and the ternary system shows substantial coincidence with some small amplitude deviations (Figure S1). The degree of meaningfulness of these deviations was assessed by calculating the rotational correlation time (*τ_c_*) of the nitroxide in the different tested conditions, according to Equations (4) and (5) (see Section 4). Table 1 lists the corresponding values. Under any tested condition, the tempol *τ_c_* value remains around an average of 31.6 ps (the standard deviation is 1.3 ps). This indicates that, within the experimental error, no detectable effect arises from β2m, or AuNPs, or both on the tumbling rate of the free radical. On the other hand, that average *τ_c_* value is consistent with those reported for 2 mM Tempo (91.9 ps) and Tempone (14.9 ps) in water at 300 K [21]. Therefore, the occurrence of Tempol interactions other than the statistical collision in the binary and ternary systems here considered should be ruled out.

**Figure 1:**
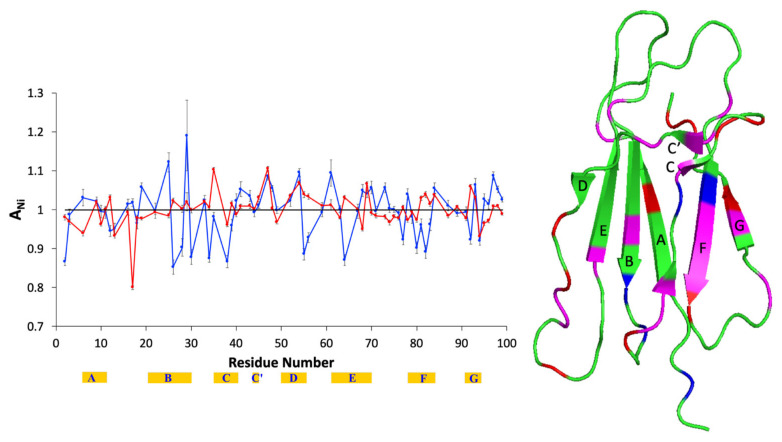
Overlay of the *A_N_* values obtained from ^1^H-^15^N HSQC spectra of 8 μM β2m in the presence of 0.8 mM Tempol, with a relaxation delay of 0.5 s (**blue**) or 5 s (**red**). The β-strand location and naming along the sequence is reported with yellow strips. The cartoon on the right highlights the positions of the accessible amides (red), i.e., exhibiting *A_N_*[eq] > 1, and the amides with Type II deviation of *A_N_*[off-eq] (**blue**), i.e., displaying *A_N_*[off-eq] < *A_N_*[eq]. The magenta color denotes sites where both *A_N_*[eq] > 1 and Type II deviation occur simultaneously. Here and elsewhere, the reproduced structure is the NMR solution structure of β2m [10] (Protein Data Bank or PDB code 1JNJ). The secondary structure elements of β2m are indicated according to the crystallographic naming scheme (PDB code 3HLA). Structures are always drawn with PyMOL (Schrödinger, Inc., version 2.3.5, New York, NY, USA).

Figure 1 shows the pattern of the normalized attenuation (*A_N_*) values observed with 8 μM β2m and 0.8 mM Tempol with respect to the same protein solution without the nitroxide. The numerical values are listed in Table S1, along with the corresponding errors. The graph of Figure 1 depicts in red the backbone amide signal *A_N_* values extracted from data collected under magnetization equilibrium conditions (*A_N_*[eq]), and in blue the analogous *A_N_* values extracted from data collected under magnetization off-equilibrium conditions (*A_N_*[off-eq]), which were respectively obtained from pairs of ^15^N-^1^H HSQC spectra acquired with relaxation delays of 5 s and 0.5 s. According to our previous interpretation [16,17,18], *A_N_*[eq] values larger or smaller than unity indicate amide signals attenuated above or below the average attenuation, respectively, and therefore, they identify molecular locations more or less accessible to the nitroxide probe, depending on the specific surface exposure. Instead, the interpretation of the *A_N_*[off-eq] values is related to their relationship with the corresponding *A_N_*[eq] figures [16]. In particular, the pattern *A_N_*[off-eq] > *A_N_*[eq] identifies amide positions with either locally hindered accessibility on the molecular surface or true structurally buried positions. As such, this pattern, which was named the Type I deviation of *A_N_*[off-eq], is typically, though not exclusively, associated with *A_N_*[eq] < 1, i.e., locations with accessibility lower than average [16]. As a matter of fact, when Type I deviation occurs at exposed locations, the corresponding *A_N_*[eq] value is only slightly larger than unity. On the other hand, the pattern *A_N_*[off-eq] < *A_N_*[eq], named Type II deviation of *A_N_*[off-eq], identifies those amide positions whose recovery is faster than the average off-equilibrium signal recovery, thereby proving less attenuated than that average. In the absence of specific interactions of the spin probe with the protein and/or structural transitions of the latter induced by the former, as verifiable by the invariance of the amide signal chemical shifts (Figure S2), Type II deviation of *A_N_*[off-eq] can be associated to local chemical or conformational exchange occurring on a ms-to-μs time scale that introduces additional relaxation increments affecting both *T*_1*p*_ and *T*_2*p*_, i.e., the paramagnetic contribution to longitudinal and transverse relaxation times [16].

The pattern of Figure 1 is different with respect to the corresponding one previously observed in diluted conditions, precisely at β2m concentration of 50 μM probed with 250 μM Tempol [16]. Apart from the larger error affecting the previous data, there are two important differences to point out. First, the former tempol/protein ratio was 5:1, whereas here, we consider a ratio of 100:1. These ratios and the absolute concentrations affect directly the collision probability [22]. We reasoned that a high tempol/protein ratio is required to balance a low absolute concentration of β2m and measure the paramagnetic perturbation. This is confirmed by the slight increase above the unity of the average relative intensity, RI_av_, (for RI definition see Section 4) under off-equilibrium conditions [16] (Figure S3).

Second, at 8 μM concentration, the extent of β2m dimerization and higher oligomerization should be further reduced compared to 50 μM [14,23,24]. Hence, it could be possible to observe features related to the protein association interface. Table 2 lists the details of the pattern observed in Figure 1 compared to the earlier results at 50 μM [16]. The most relevant differences concern the higher exposure in the 8 μM solution of strands C, D, F, and G and the intensification in local conformational or chemical exchange at strands C and F, with a simultaneous loss of accessibility at strand A and loop AB. In addition to the relevance for the involvement in the association interface, these features are also important to delineate a starting point and thus appreciate the interactions and structural effects that the presence of AuNPs may induce.

**Figure 2:**
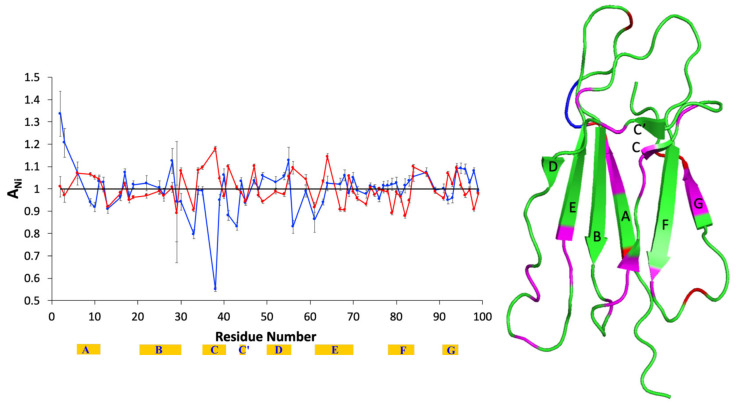
Overlay of the *A_N_* values obtained from ^1^H-^15^N HSQC spectra of 8 μM β2m + 60 nM gold nanoparticles (AuNPs) in the presence of 0.8 mM Tempol, with a relaxation delay of 0.5 s (**blue**) or 5 s (**red**). The β-strand location and naming along the sequence is reported with yellow strips. The cartoon on the right highlights the positions of the accessible amides (**red**), i.e., exhibiting *A_N_*[eq] > 1, and the amides with Type II deviation of *A_N_*[off-eq] (**blue**), i.e., displaying *A_N_*[off-eq] < *A_N_*[eq]. The magenta color denotes sites where both *A_N_*[eq] > 1 and Type II deviation occur simultaneously. The secondary structure elements of β2m are indicated according to the crystallographic naming scheme (PDB code 3HLA).

The same paramagnetic perturbation analysis as done with isolated β2m can be performed for the system protein + AuNPs, because a statistical collision model can still be adopted, according to the ESR-based determinations of the Tempol *τ_c_* values under different experimental conditions. Moreover, AuNPs are known to essentially preserve the chemical shifts, and therefore the structure, of β2m and D76N β2m [2,4,6], although at concentrations and NP/protein ratios as low as 4–8 μM and 1/100–1/200, small chemical shift deviations have been detected for both variants [2,6]. Most of these deviations were observed with synthetic AuNPs with an average diameter of 7.5 ± 1 nm, and therefore, some difference can be expected upon decreasing the NP diameter to 5 nm. With the commercial AuNPs here employed, minor chemical shift perturbations [25] are measured at Q2, N17, S33, D38, and S61 NHs of β2m, which in two cases (N17, D38) decrease below the resolution significance in the presence of Tempol (Figure S2). A possible explanation for those chemical shift perturbations may be related to NP-induced alterations of the intra-residue interaction between the side-chain polar group and the backbone amides. From the results reported in Figure 2 and Table 3, it can be seen that N17 and D38 become accessible to Tempol in the presence of AuNPs. Therefore, the reduction of their chemical shift perturbation could be related to the interaction with Tempol that, albeit non-specific, competes with the intra-residue interaction. On the other hand, the conserved chemical shift deviations of Q2, S33, and S61 after Tempol addition match with a hindered accessibility in the presence of AuNPs (Figure 2, Table 3). Therefore, all of the observed chemical shift perturbations can be attributed exclusively to the protein interaction with the NPs. However, given the substantial invariance of the fingerprint pattern in the ^15^N-^1^H HSQC spectra and the limited amounts of the frequency changes, all the mentioned deviations do not impair the assumptions of protein structure conservation and statistical character for the nitroxide probing. However, all the previous evidence indicates that AuNPs unevenly affect the intensity of the ^15^N-^1^H HSQC peaks of β2m and variants thereof [2,3,4,5,6,7]. Although this effect is precious to interpret the molecular details of the protein interaction with nanoparticles, it may prove detrimental when evaluating the paramagnetic attenuation contributed by the nitroxide probe, under magnetization equilibrium or off-equilibrium conditions.

A preliminary control of the ^15^N longitudinal relaxation rates is useful to estimate the entities of the effects brought about by AuNPs and Tempol on β2m signals (Figure S4). Indeed, the average *T*_1_ value of 8 μM β2m decreases by the same extent, i.e., 4%, with AuNPs or Tempol, whereas the addition of the nitroxide to β2m in AuNPs suspension shortens the average *T*_1_ only by 0.7%. This means that the Tempol paramagnetic attenuation probing the statistical sampling differences would be extensively masked by the general and specific dipolar attenuation AuNPs inflict to β2m signals, if the *A_N_* values were computed with respect to the isolated protein intensities. In conclusion, proper control intensities should be obtained from the protein sample in the presence of AuNPs rather than from the protein alone. Figure 2 shows the attenuation pattern of the backbone amide signals observed with 8 μM β2m and 60 nM AuNPs due to 0.8 mM Tempol. The numerical values are listed in Table S2, along with the corresponding errors. According to the preliminary *T*_1_ control measurements, the *A_N_* values are calculated with respect to the control solution, i.e., the sample with the same composition without the nitroxide. Compared to the *A_N_* profile of the isolated protein (Figure 1), evident differences emerge in the *A_N_* plot of Figure 2 concerning exposed or poorly accessible regions, as well as locations undergoing chemical or conformational exchange. In particular, AuNPs hinder accessibility at the N-terminal, end of strand B, loop CC’ and subsequent strand C’, start of strand D, strand F, and C-terminal of β2m, while increasing strand A exposure. The details of these differences can be appreciated from Table 3, which lists the equilibrium and off-equilibrium attenuation data with (bold fonts) and without (plain fonts) AuNPs, thereby enabling a direct comparison.

## 3. Discussion

The results above described must be analyzed from two viewpoints. On one side, the nitroxide-based screening on the free protein helps to gather elements on the association processes β2m undergoes in solution. These elements may reveal features related to the fibrillogenic propensity of β2m. On the other side, the same paramagnetic probing of the protein in the AuNPs suspension provides the elements that enable outlining the β2m interaction with those nanoparticles. Joining these two lines of evidence is particularly tempting, because it is thus possible to focus the mechanism of fibrillogenesis inhibition experimentally observed with AuNPs and the β2m variant D76N [4].

As pointed out in the previous section, the most relevant differences highlighted by equilibrium and off-equilibrium nitroxide collisional labeling when comparing the 8 μM and the 50 μM β2m solutions concern the higher exposure in the former of strands C, D, F, and G and the intensification in local conformational or chemical exchange processes at strands C and F, with a simultaneous loss of accessibility at strand A and loop AB (Table 2). This result is consistent with the previous inference and evidence addressing respectively the intermolecular interface in ∆N6 β2m, the variant devoid of the N-terminal hexapeptide fibrils [14], and the H-D exchanged β2m fibrils dissolved in DMSO [26]. In particular, it is very meaningful that the increased accessibility at strands C and F observed at lower concentration is paralleled by the onset, at the same positions, of exchange events (*A_N_*[off-eq] Type II deviations, Table 2) that must witness the remnant of the intermolecular interaction propensity at those locations. At higher concentration, the pattern changes into significantly lower exposure and exchange due to the shift of the association dynamics toward oligomeric adducts.

**Figure 3:**
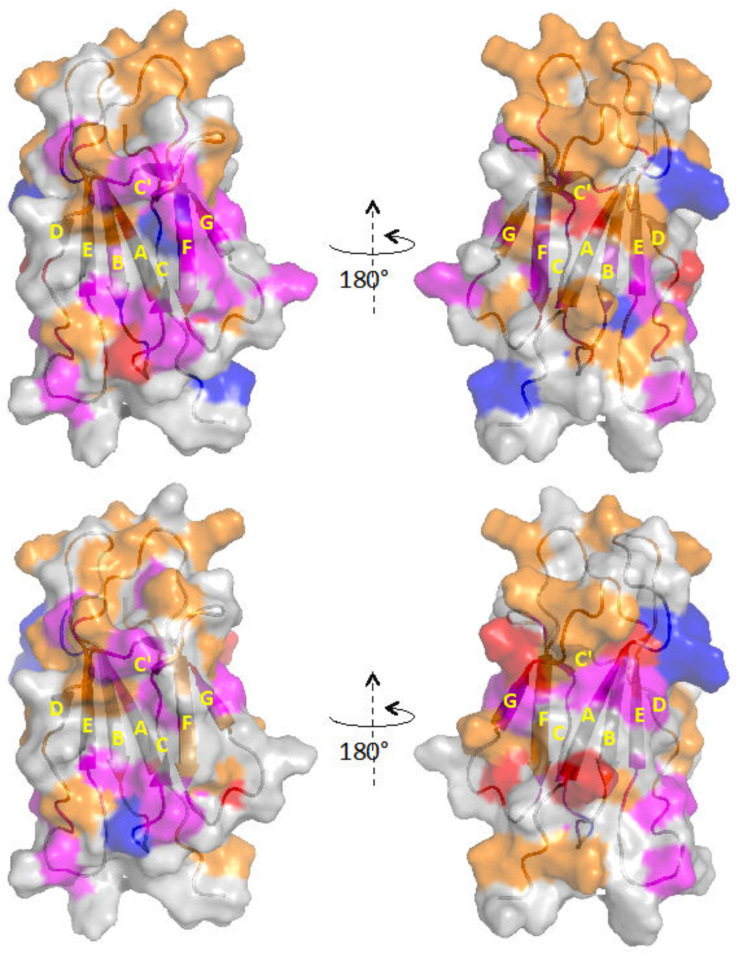
Cartoon representation of β2m surface sampled by Tempol in the absence (**upper pair**) and presence (**lower pair**) of AuNPs. The positions of the accessible backbone amides, i.e., exhibiting *A_N_*[eq] > 1, are marked in red. The locations of the amides with Type I or Type II deviation of *A_N_*[off-eq], i.e., displaying *A_N_*[off-eq] > *A_N_*[eq] or *A_N_*[off-eq] < *A_N_*[eq], are highlighted in orange or blue, respectively. The magenta color denotes sites where both *A_N_*[eq] > 1 and Type II deviation occur simultaneously. The very few positions where *A_N_*[eq] > 1 and Type I deviation coincide were left in orange. The secondary structure elements of β2m are indicated according to the crystallographic naming scheme (PDB code 3HLA).

The statistical sampling of Tempol shows that the presence of AuNPs exposes strand A and limits accessibility at the very N-terminal segment, end of strand B, turn CC’ and strand C’, start of strand D, strand F, and C-terminal region of β2m. Again, the gain or loss of accessibility at strands A, B, C’, and F, and turn CC’ are accompanied, at the same locations, by a gain or loss of Type II deviation of *A_N_*[off-eq]. However, except for the N-terminal fragment (Q2, R3), the interaction pattern obtained by paramagnetic perturbation does not seem to match the previously reported picture based on relative intensity losses affecting essentially the end of strands B and D, and loops BC and DE [2]. The differences in β2m concentrations (8 vs. 26 μM) and protein/AuNPs ratios (100 vs. 200) between present and previous experiments could certainly play a role in determining some deviations. However, the relative intensity changes marking the difference between the absence and presence of AuNPs, and the paramagnetic perturbation measured in the presence of AuNPs, monitor different aspects of β2m interaction with AuNPs, and thus, the mismatch may be only apparent. In fact, similar to the previously reported low relative intensity of Q2 and R3 induced by AuNPs that translates into a Type I deviation of *A_N_*[off-eq], i.e., hindered accessibility to the nitroxide probe, a similar effect with the paramagnetic perturbation is seen also for S28 (strand B, see Table 3). More frequently, instead, the β2m residues reported to lower the relative intensities due to AuNP interaction [2] exhibit Type II deviation of *A_N_*[off-eq], i.e., local exchange, with nitroxide probing in the presence of AuNPs, which are typically coupled to above average accessibility (*A_N_*[eq] > 1). This is the case with the ends of strands B and D, and with the involved subsequent locations at BC and DE loops (Table 3). Therefore, the picture emerging from the interpretation of the results obtained by equilibrium and off-equilibrium paramagnetic mapping does not conflict with the previous evidence but rather provides a more detailed and enriched characterization of the interaction between β2m and AuNPs. This appears quite clearly by the inspection of Figure 3 where the surface of the protein according to the equilibrium and off-equilibrium attenuation pattern induced by Tempol in the absence (upper structures) and presence (lower structures) of AuNPs is highlighted.

Hindered accessibility is marked by orange surfaces that change their distribution on moving from the isolated protein to the presence of AuNPs. In either conditions, those surfaces could be associated to regions that become screened by the relevant interaction, namely the residual protein-protein or the protein-nanoparticle one. The occurrence of exchange processes at the blue and magenta locations can be considered the consequence of an interaction that takes place over the ms-to-µs time scale and may represent a further interface with different dynamic properties with respect to the hindered accessibility surface, provided that the occurrence of a local conformational exchange process is ruled out [16]. Finally, the highly accessible positions that are identified in red indicate the surface that is not involved in any protein-protein nor protein-nanoparticle contact. Based on the discussed evidence for β2m alone [14,26] and with AuNPs [2,3], the results listed in Table 2 and Table 3 and depicted in Figure 3 suggest that the AuNP interference leading to the inhibition of fibrillogenesis [4,6] could occur via interaction of the nanoparticles with the N-terminal and strands D and F of β2m, in addition to other contacts at the end of strand B, turn CC’, and strand C’. The suggested hypothesis is that these interactions in which AuNPs engage with the protein surface prevent the protein-protein contacts at the same locations that are necessary for fibrillogenic aggregation.

## 4. Materials and Methods

### 4.1. Chemicals

Sodium Citrate, ^2^H_2_O, Tempol (4-hydroxy-2,2,6,6-tetramethyl-piperidine-l-oxyl), and HEPES (*N*-(2-Hydroxyethyl)piperazine-*N*’-(2-ethanesulfonic acid) were all from Sigma Aldrich (St. Louis, MO, USA). From the same source were also the citrate-stabilized Au nanoparticles, here referred to as AuNPs. The average AuNP diameter was 5 nm, and the supplied suspension concentration was 91 nM.

### 4.2. Sample Preparation

The uniformly ^15^N-labeled wild-type human β2m was expressed with an additional methionine at the N-terminus (Met-0) and purified as previously reported [14]. The protein samples in the absence of AuNPs were prepared in H_2_O/D_2_O 95/5, 1.5 mM sodium citrate, 20 mM HEPES buffer, pH 7. The protein concentration was 8.0 µM, as determined by UV absorption at 280 nm. For solutions with AuNPs, proper amounts of D_2_O and concentrated HEPES and β2m solutions were added to the mother NP suspension containing already citrate to reproduce the above-mentioned composition. Following dilution, the final AuNP concentration was 60 nM. A few microliters of concentrated Tempol solution were added when necessary to the NMR tube containing 0.550 mL of β2m, with or without AuNPs, to reach the desired Tempol/protein concentration ratio. For NMR samples, Tempol concentration was always 0.8 mM. For ESR samples, solutions at variable Tempol concentrations were prepared, i.e., 0.4, 0.8, and 1.6 mM, in aqueous buffer (20 mM HEPES, 1.5 mM sodium citrate, pH = 7), either alone or in the presence of 8 µM β2m, or 60 nM AuNP, or 8 µM β2m + 60 nM AuNP.

### 4.3. Spectroscopy

All the spectra were acquired at 298 K. The NMR experiments were collected at 14.0 T (^1^H at 600.19 MHz, ^15^N at 60.82 MHz) on a Bruker Avance III NMR system equipped with triple resonance cryoprobe. Two-dimensional ^15^N-^1^H HSQC experiments [27] carried out using sensitivity-improved Echo/Antiecho-TPPI pure phase detection in F1, gradient coherence selection, and flip-back pulse for solvent suppression [28,29,30] were acquired over spectral widths of 40 ppm and 14 ppm in F1 and F2 dimensions, respectively, with 64 time-domain points in t1, 256 or 512 scans × 2048 points in t2, and 64 dummy scans to achieve steady state. After a reproducibility check, relaxation delays were set to 0.5 and 5 s, respectively for off- and on-equilibrium conditions of magnetization recovery, following the guidelines previously reported [16]. The contour plots are reported in Figure S5, along with the corresponding signal-to-noise values (Table S3). The ^15^N longitudinal relaxation times were measured using the sequence proposed by Kay and colleagues [31] with the modifications for sensitivity enhancement and flip-back pulse for solvent suppression [28,29,30]. The spectra with eight different relaxation intervals were acquired (10, 30, 60, 100, 140, 200, 400, and 1200 ms). All NMR data were processed with TOPSPIN version 4.0.2. Prior to Fourier transformation, linear prediction in t1 (up to 128 points) and zero filling were applied to yield a final data set of 2 K × 1 K points. For longitudinal relaxation analysis, the Bruker Dynamics Center 2.5.3 routine was used.

ESR spectroscopy experiments were collected with a Bruker EMXnano spectrometer operating in the X band. Capillaries filled with 50 μL of sample solution were placed in standard 4 mm tubes and submitted to acquisition (1 scan). The ESR operating parameters were as follows: frequency = 9.6 GHz; microwave power = 0.316 mW; modulation amplitude = 1 Gauss; modulation frequency = 100 kHz; center field = 3429.8 Gauss; sweep width = 200 Gauss; time constant = 1.28 ms. The data were processed using the software package Xenon (version 1.1b50, Bruker, Billerica, MA, USA).

### 4.4. Spectroscopic Data Treatment

Amide cross-peak intensities in ^15^N-^1^H HSQC spectra of β2m in the absence (*I_d_*) and in the presence (*I_p_*) of Tempol or/and AuNPs were measured by SPARKY software (version 3.133, T.D. Goddard and D.G. Kneller, University of California, San Francisco CA, USA). Normalized attenuation, *A_N_*, was calculated according to Equation (1) [18]

(1) $$A_{N}^{k}=\left(2-\frac{{ı}_{p}^{k}}{{ı}_{d}^{k}}\right)\;$$

where the running index *k* refers to the kth residue amide cross-peak and the $\iota_{p,d}^{k}$ values are the corresponding auto-scaled intensities of the peaks in the presence (subscript *p*) and absence (subscript *d*) of nitroxide or/and AuNPs, which are defined as

(2) $${ı}_{p,d}^{k}=\frac{I_{p,d}^{k}}{\frac{1}{n}\sum_{k=1}^{n}I_{p,d}^{k}}\;$$

with *n* representing the total number of measured peaks. From the above equation, it is seen that the scaling factor is simply the mean value over the *n* molecular locations for which the corresponding peak intensity can be estimated ($I_{p,d}^{av}$), the mean value of the individual auto-scaled intensities being unitary, by definition [16,18]. Therefore, values of *A_N_* above or below unity indicate larger or smaller attenuations, respectively, with respect to the average absolute signal attenuation. From the definitions, the error on the individual *A_N_* values can be calculated as [16]

(3) $$∆A_{N}^{k}=A_{N}^{k}\times \sqrt{{\left[\frac{∆I_{p}^{k}}{I_{p}^{k}}\right]}^{2}+{\left[\frac{∆I_{d}^{k}}{I_{d}^{k}}\right]}^{2}+\left[\frac{\frac{1}{n^{2}}\sum {\left(∆I_{p}^{k}\right)}^{2}}{{\left(I_{p}^{av}\right)}^{2}}\right]+\left[\frac{\frac{1}{n^{2}}\sum {\left(∆I_{d}^{k}\right)}^{2}}{{\left(I_{d}^{av}\right)}^{2}}\right]}$$

where the first two terms under the square root sign represent the error on the relative intensity (*RI*) of the *k*th residue signal, i.e., the signal intensity ratio in the presence and absence of nitroxide or/and AuNPs, and the ∆*I* are the experimental intensity uncertainties obtained from the individual peak signal-to-noise figure.

The ESR spectra were employed to extract the rotational correlation time (*τ_c_*) of Tempol in absence or presence of β_2_m or/and AuNPs. Based on the method of Knowles and colleagues [32] and Kivelson’s theoretical analysis [33], the *τ_c_* values were estimated from:

*τ_c_* = 6.5 × 10^−10^ ∆*B*_0_[(*h*_0_/*h*_−1_)^1/2−1^] (4)

where ∆*B*_0_ is the linewidth of the of the central line of the nitroxide ESR signal (a triplet because of the hyperfine coupling with the ^14^N nuclear spin), and *h*_0/−1_ are the amplitudes of the central and upfield lines. The corresponding error was calculated from Equation (4) by error propagation of the experimental uncertainties on linewidth (∆∆*B*_0_) and amplitudes (∆*h*_0/−1_), according to:

(5) $$∆\tau_{c}=6.5\cdot 10^{-10}\left\{\left[{\left(\frac{h_{0}}{h_{-1}}\right)}^{\frac{1}{2}}-1\right]∆\Delta B_{0}+\frac{\Delta B_{0}}{2{\left(h_{-1}\right)}^{\frac{1}{2}}{\left(h_{0}\right)}^{\frac{1}{2}}}∆h_{0}+\frac{\Delta B_{0}{\left(h_{0}\right)}^{\frac{1}{2}}}{2{\left(h_{-1}\right)}^{\frac{3}{2}}}∆h_{-1}\right\}$$

## 5. Conclusions

The results here described demonstrate that the paramagnetic perturbation methodology can be successfully applied to study protein-nanoparticle interactions. In addition to the surface accessibility mapping, extrinsic paramagnetic probes can provide valuable information on hindered accessibility and exchange processes by means of off-equilibrium attenuation analysis [16]. This methodology represents an additional tool that enriches the NMR relaxation approach to the characterization of protein interaction with the nanoparticle surface [34]. The delineation of the contact interface between protein monomers and between protein and nanoparticles is important not only for the comprehension of the mechanisms of protein aggregation and the elaboration of contrast strategies that bear particular relevance in amyloidogenic systems, but also for the characterization of that ensemble of labile contacts that is involved in the build-up of the so-called soft corona, i.e., the coating layer of weakly bound protein molecules with short residence times that can affect the nanoparticle targeting [35].

## Figures and Tables

**Table 1:** Rotational correlation time (*τ_c_*_/_10^−11^ s) of Tempol at the indicated concentrations and different solution compositions obtained from ESR measurements at 298 K.

Composition	Tempol	Tempol + AuNPs	Tempol + β2m	Tempol + AuNPs + β2m
[Tempol]		[AuNP] = 60 nM	[β2m] = 8 μM	[AuNP] = 60 nM; [β2m] = 8 μM
1.6 mM	3.3 ± 0.2	3.1 ± 0.2	3.4 ± 0.2	3.3 ± 0.2
0.8 mM	3.2 ± 0.2	3.0 ± 0.3	3.2 ± 0.3	2.9 ± 0.2
0.4 mM	3.1 ± 0.3	3.2 ± 0.3	3.1 ± 0.3	3.1 ± 0.4

**Table 2:** Paramagnetic perturbation induced by 0.8 mM Tempol on the amide NMR signals of 8 μM β2m. Equilibrium (column 2) and off-equilibrium (columns 3 and 4) data are compared to the corresponding data obtained at 50 μM β2m and 5:1 Tempol:protein ratio [16] and reported below in italic fonts.

Structure Region	A_N_ > 1	Type I A_N_ [off-eq] > A_N_ [eq]	Type II A_N_ [off-eq] < A_N_ [eq]
N-term, strand A	V9 *Q2, I7*	K6, Y10	Q2 *Q2, I7*
loop AB	*R12, K19*	H13, E16, N17, K19 *N17*	R12 *R12, H13*
strand B	Y26	C25, G29 *S28*	Y26, S28, F30 *N21, F22, C25, F30*
loop BC	S33, D34 *D34*	*S33*	D34 *D34*
strand C	I35, L39, K41 *I35*	L40, K41	I35, D38, L39 *I35*
turn CC’, strand C’, loop C’D	G43, R45, E47 *G43, E47, K48, V49*	G43, K48, V49 *R45, K48*	R45, E47 *N42*
strand D	S52, L54, S55, F56 *F56*	L54 *E50, L54*	S55, F56 *S52, F56*
loop DE	D59	S61 *D59*	*S61*
strand E	L64, E69 *E69*	Y63, T68, F70 *E69, F70*	L64, E69 *L64*
loop EF	E77 *K75*	T73, E74, K75, D76 *T71, E74*	E77
strand F	R81, V82, N83, H84 *N83, H84*	T78 *T78*	C80, R81, V82, N83 *A79, H84*
loop FG	Q89 *V85, L87*	L87	Q89 *V85*
strand G, C-term	I92, V93, R97, D98 *K94*	V93, W95, D96, R97, D98, M99 *V93, D96, D98, M99*	I92 *I92, K94*

**Table 3:** Paramagnetic perturbation induced by 0.8 mM Tempol on the amide NMR signals of 8 μM β2m with 60 nM AuNPs. The equilibrium (column 2) and off-equilibrium (columns 3 and 4) data are reported in bold. The corresponding data obtained without AuNPs (Table 2) are reproduced below in plain fonts.

Structure Region	A_N_ > 1	Type I A_N_ [off-eq] > A_N_ [eq]	Type II A_N_ [off-eq] < A_N_ [eq]
N-term, strand A	**K6, V9, Y10, S11** V9	**Q2, R3** K6, Y10	**V9, Y10** Q2
loop AB	**N17**	**N17, K19** H13, E16, N17, K19	**R12, H13** R12
strand B	**F30** Y26	**F22, S28** C25, G29	**F30** Y26, S28, F30
loop BC	**D34** S33, D34		**S33, D34** D34
strand C	**I35, D38, L39, K41** I35, L39, K41	**L40** L40, K41	**I35, D38, L39, K41** I35, D38, L39
turn CC’, strand C’, loop C’D	**E47** G43, R45, E47	**E44, K48, V49** G43, K48, V49	**E47** R45, E47
strand D	**S55, F56** S52, L54, S55, F56	**S52, L54** L54	**S55, F56** S55, F56
loop DE	**D59** D59	S61	**D59**
strand E	**Y63, L64, E69** L64, E69	**Y67, T68, F70** Y63, T68, F70	**Y63, L64, E69** L64, E69
loop EF	E77	**T71, T73, K75** T73, E74, K75, D76	E77
strand F	**H84** R81, V82, N83, H84	**A79, C80, V82, N83** T78	**H84** C80, R81, V82, N83
loop FG	**L87** Q89	L87	Q89
strand G, C-term	**I92, V93, K94, W95** I92, V93, R97, D98	**K91, W95, D96, R97, D98** V93, W95, D96, R97, D98, M99	**I92, V93** I92

## Supplementary Materials

The following are available online, Figure S1: overlay of ESR spectra; Figure S2: chemical shift perturbation values of β2m amide signals under different conditions; Figure S3: relative intensity plots of 8 µM + 0.8 mM Tempol under magnetization equilibrium and off-equilibrium conditions; Figure S4: ^15^N longitudinal relaxation times of β2m amide signals under different conditions. Figure S5: ^15^N-^1^H HSQC maps; Table S1: *A_N_* values plotted in Figure 1 and corresponding errors; Table S2: *A_N_* values plotted in Figure 2 and corresponding errors; Table S3: individual peak signal-to-noise ratios of ^15^N-^1^H HSQC spectra.
