# Supplementary material for: Insights into a Protein-Nanoparticle System by Paramagnetic Perturbation NMR Spectroscopy
Source: Molecules. 2020 Nov 7;25(21):5187. doi: 10.3390/molecules25215187 (PMC7664681; doi:10.3390/molecules25215187)
Supplement: Supplementary file 1 [file molecules-25-05187-s001.pdf]

Insights into a protein-nanoparticle system by paramagnetic perturbation

Yamanappa Hunashal[1,2], Cristina Cantarutti[3], Sofia Giorgetti[4], Loredana Marchese[4], Federico Fogolari[5,6], and Gennaro Esposito[1,6,*]

[1]Science Division, New York University Abu Dhabi, Abu Dhabi – UAE.
[2]DAME, Università di Udine, P.le Kolbe, 4, 33100 Udine, Italy.
[3]Institute de Chimie, UMR CNRS 7272, Université Côte d'Azur, Université de Nice Sophia Antipolis, Parc Valrose, 06108, Nice Cedex 2, France.
[4]Dip. Medicina Molecolare, Università di Pavia, Via Taramelli 3, 27100 Pavia, Italy.
[5]DMIF, Università di Udine, Viale delle Scienze, 33100 Udine, Italy.
[6]INBB, Viale Medaglie d'Oro 305, 00136 Roma, Italy.

* Correspondence: rino.esposito@nyu.edu

SUPPLEMENTARY MATERIALS

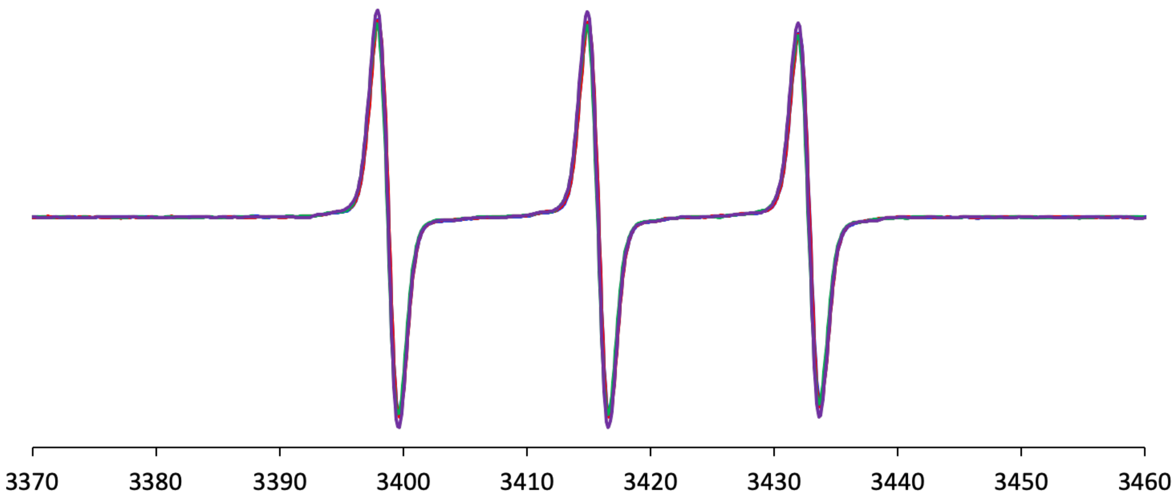

Figure S1. Superposition of the ESR spectra of 0.8 mM Tempol (blue), 0.8 mM Tempol + 8 µM β2m (red), 0.8 mM Tempol + 60 nM AuNPs (green) and 0.8 mM Tempol + 8 µM β2m + 60 nM AuNPs (purple). All solutions were prepared in 20 mM Hepes at pH 7. For the samples without AuNPs, 1.5 mM sodium citrate (already present in the AuNPs preparations) was added to the solvent. The spectra were recorded at 298 K. The barely detected amplitude differences are consistent with rotational correlation times with differences within the experimental error (see Table 1 of main text).

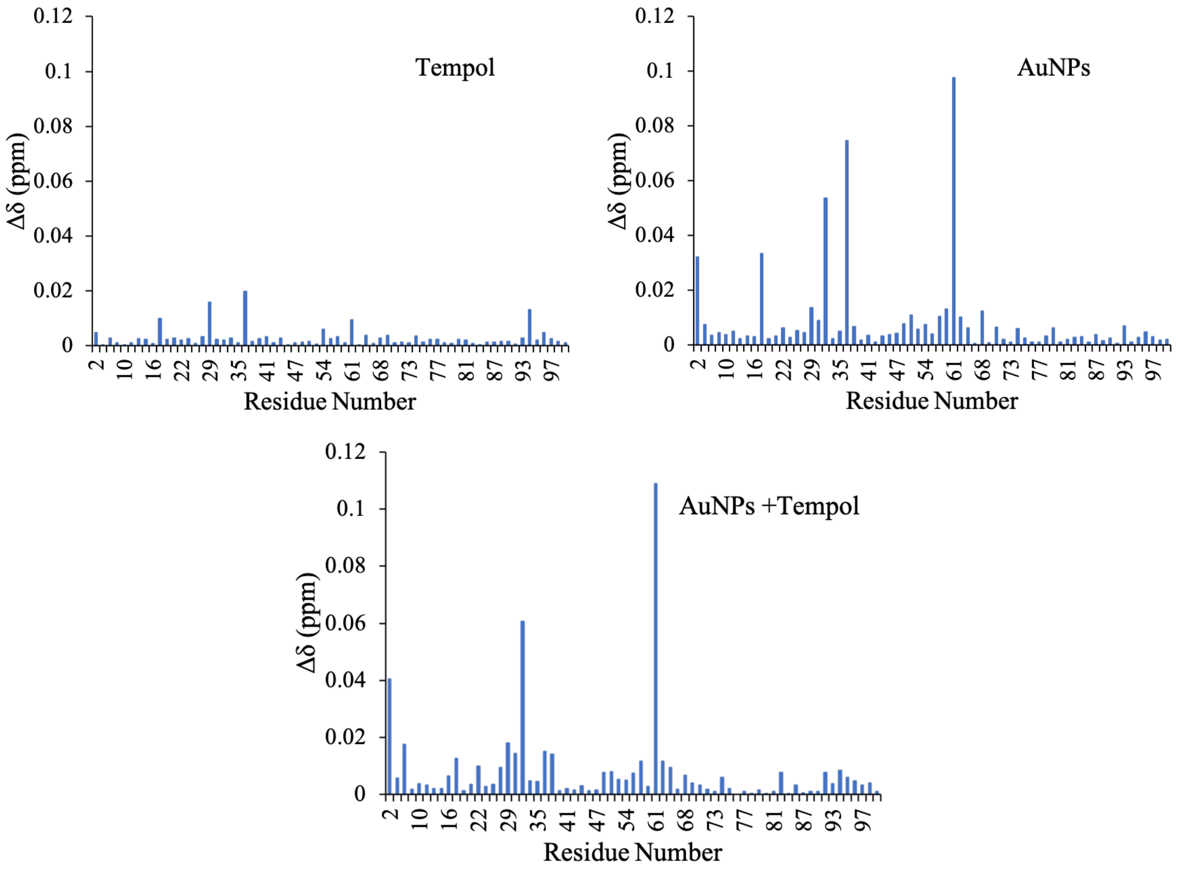

Figure S2. Chemical shift perturbation (CSP) of β2m backbone amide signals induced by Tempol (0.8 mM, protein/Tempol = 0.01) or AuNPs (60 nM, protein/AuNPs = 133) or AuNPs + Tempol (at the same concentrations and concentration ratios) in ^{15}N-^{1}H HSQC spectra.

To properly account for both ^{1}H ($\Delta\delta_H$) and ^{15}N ($\Delta\delta_N$) frequency changes, the chemical shift variations ($\Delta\delta$) of the protein amide peaks from ^{15}N-^{1}H HSQC spectra in the presence of nitroxide or/and AuNPs were analyzed in terms of cumulated CSP, according to:

$$CSP = \sqrt{\Delta\delta_H^2 + \left(\frac{\Delta\delta_N}{6.5}\right)^2}$$

as previously reported (reference [25] of main text).

Only CSP values above 0.02 ppm are considered meaningful (the limit should be increased to 0.05 ppm if the acquisition resolution is considered). This is the case of Q2, N17, S33, D38 and S61, in the presence of AuNPs, and Q2, S33 and S61 with AuNPs and Tempol. For the sample with Tempol only, all CSP are below the resolution significance.

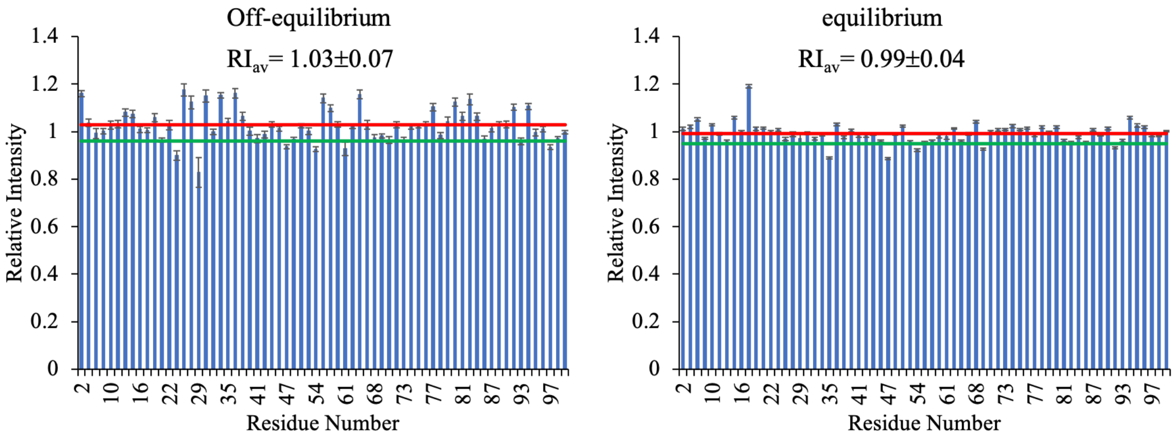

Figure S3. Relative intensities (RI) from ^{15}N-^{1}H HSQC spectra of β2m amide signals measured for the mixture of 8 μM protein and 0.8 mM Tempol. The RI values are the ratios on the peak heights in the presence and absence of the nitroxide. The experimental data are collected under magnetization equilibrium and off-equilibrium conditions

The average RI value and the standard deviation ($RI_{av} \pm SD$) are given above the histograms and graphically indicated by red and green horizontal lines, respectively. It is worth noting that the equilibrium and off-equilibrium RI_{av} values are below and above 1, respectively, which is the signature of sufficient collision probability, i.e. sufficient concentrations and protein/nitroxide ratio (reference [16] of main text).

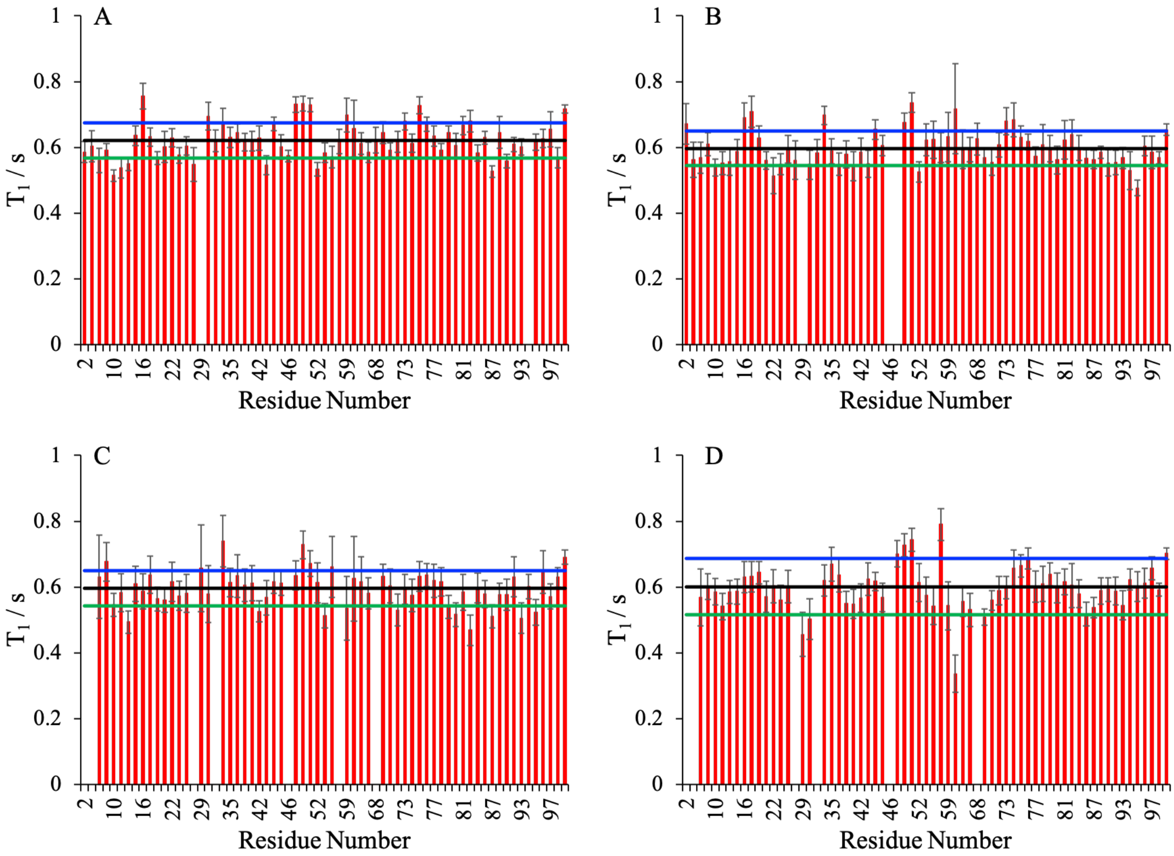

Figure S4. ^{15}N longitudinal relaxation times (T_1) of resolved backbone amide signals of β2m. The average T_1 value and the displacements by ± SD (standard deviation) are represented as black, blue and green horizontal lines. A) 8 µM β2m, average T_1 = 0.621±0.054 s. B) 8 µM β2m + 0.8 mM Tempol, average T_1 = 0.597±0.052 s. C) 8 µM β2m + 60 nM AuNPs, average T_1 = 0.596±0.054 s. D) 8 µM β2m + 0.8 mM Tempol + 60 nM AuNPs, average T_1 = 0.592±0.063 s.

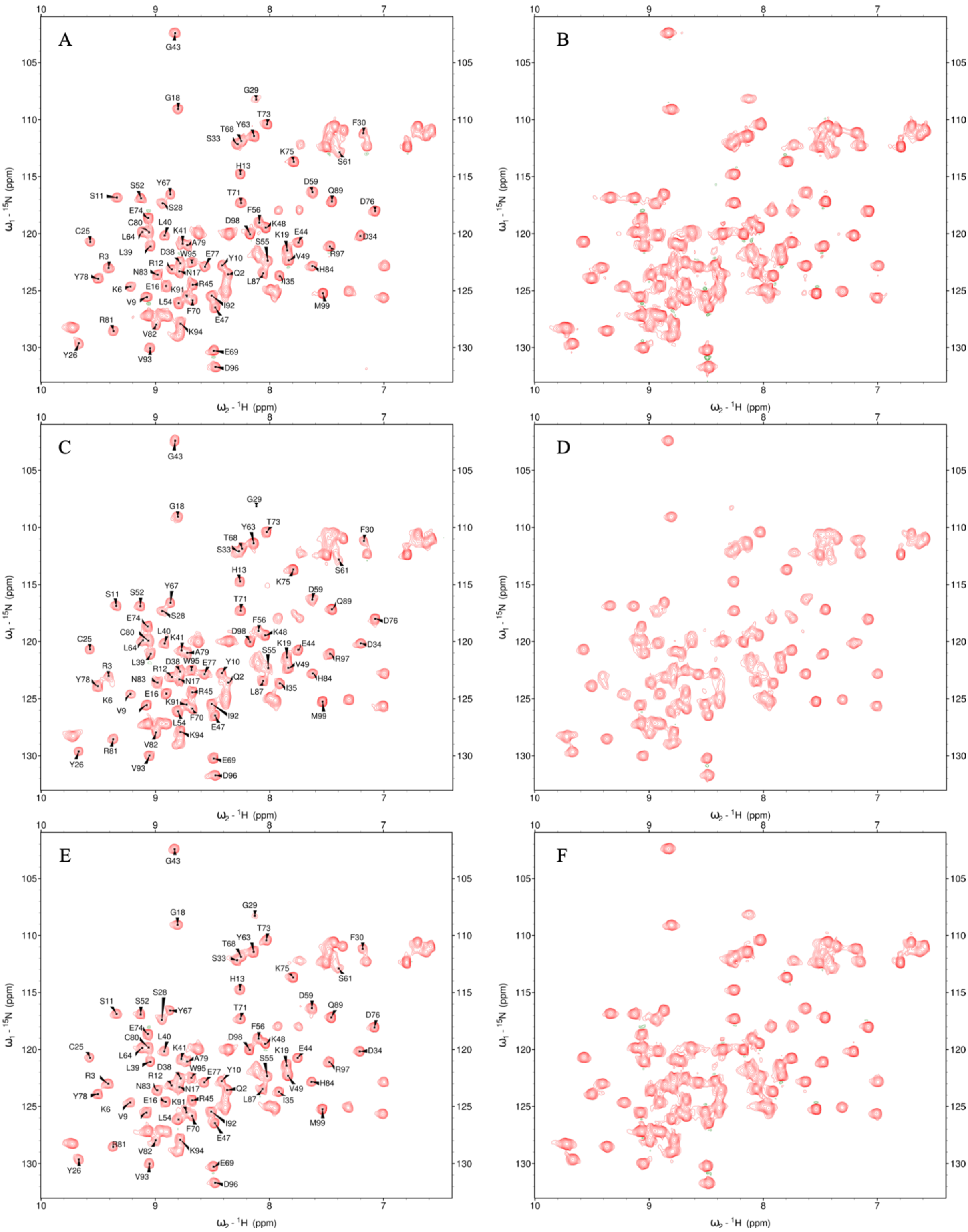
A
B
C
D
E
F
ω1 - 15N (ppm)
ω2 - 1H (ppm)

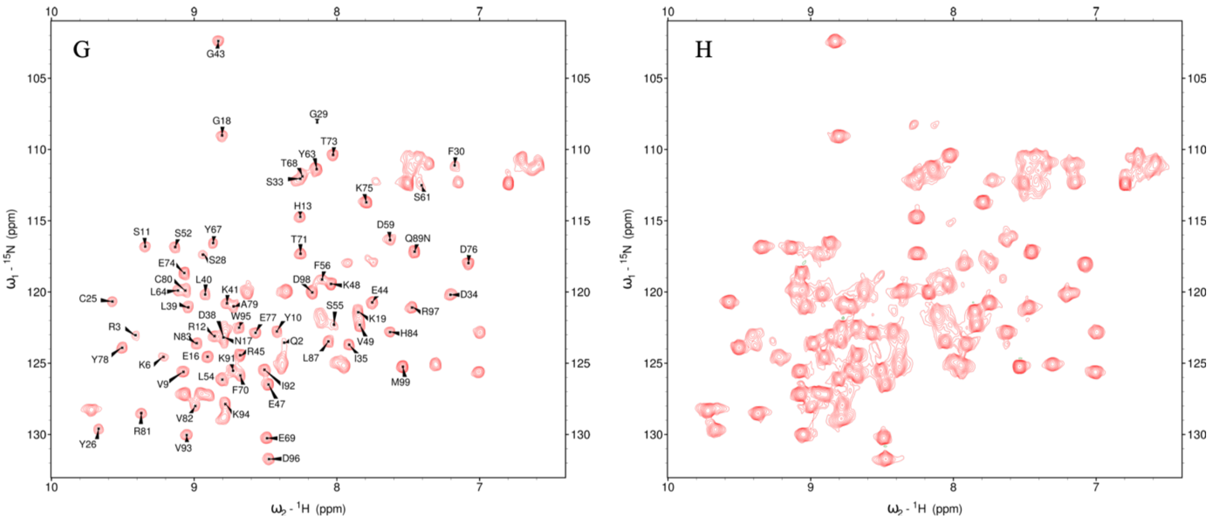

Figure S5: ^{15}N-^{1}H HSQC spectra of:

8 μM β2m obtained with relaxation delay of 0.5 s (A) and 5 s (B);

8 μM β2m + 60 nM AuNPs obtained with relaxation delay of 0.5 s (C) and 5 s (D);

8 μM β2m + 0.8mM Tempol obtained with relaxation delay of 0.5 s (E) and 5 s (F);

8 μM β2m + 60 nM AuNPs + 0.8mM Tempol obtained with relaxation delay of 0.5 s (G) and 5 s (H).

Table S1: A_N values obtained from ^{15}N-^{1}H HSQC spectra of 8 μM β2m in presence of 0.8 mM Tempol, with relaxation delay of 0.5 s and 5 s with corresponding errors calculated using Eq. 3 of main text.

Residue	***ANi*_0.5s**	**Error_0.5s**	***ANi*_4s**	**Error_4s**
Q2N-H	0.86699213	0.00986103	0.98102695	0.00708739
R3N-H	0.98735275	0.01438475	0.96990125	0.00692724
K6N-H	1.0312326	0.02167996	0.93968159	0.00710829
V9N-H	1.02163041	0.01160638	1.02045805	0.00534486
Y10N-H	0.99595924	0.01615651	0.96283927	0.00486607
S11N-H	0.99342892	0.01575729	1.00026327	0.005846
R12N-H	0.94536048	0.01462794	1.03180894	0.00600281
H13N-H	0.95146057	0.01376485	0.93219287	0.0048813
E16N-H	1.01449033	0.01203714	0.993078	0.00650459
N17N-H	1.01855706	0.01005916	0.79977757	0.00472303
G18N-H	0.96568513	0.01512453	0.97972476	0.00626468
K19N-H	1.05832865	0.01064751	0.97765334	0.00455672
F22N-H	0.9973571	0.01990403	0.99378369	0.00614801
C25N-H	1.12231697	0.02464646	0.98436121	0.00554214
Y26N-H	0.85269836	0.01832467	1.02302323	0.00633442
S28N-H	0.90299936	0.02225538	1.00238763	0.00921617
G29N-H	1.19081567	0.09068189	1.02070652	0.02348401
F30N-H	0.87799995	0.01915064	0.99833046	0.00580234
S33N-H	1.02488995	0.01169889	1.02085071	0.0063304
D34N-H	0.87458387	0.00912819	1.00615319	0.00427804
I35N-H	0.98191495	0.01157767	1.10372246	0.00524001
D38N-H	0.86653645	0.01482497	0.96148695	0.00549724
L39N-H	0.96002432	0.01510833	1.0158238	0.00648956
L40N-H	1.0229056	0.01785369	0.98764199	0.00561905
K41N-H	1.05294791	0.01866195	1.00906301	0.00579226
G43N-H	1.03556884	0.01416013	1.00973464	0.00614544
E44N-H	0.99368503	0.01109832	1.00180076	0.00433689
R45N-H	1.01198454	0.00924517	1.03196276	0.00544492
E47N-H	1.08637674	0.01072194	1.10595361	0.00582721
K48N-H	1.05452048	0.00831272	1.00379816	0.004476
V49N-H	0.99884115	0.00892593	0.96840832	0.00410225
S52N-H	1.02297721	0.01451342	1.03557721	0.0059113
L54N-H	1.09572396	0.01061915	1.06957573	0.00638364
S55N-H	0.88578307	0.01438208	1.03948117	0.00786724
F56N-H	0.92847597	0.01225593	1.03391815	0.00673705
D59N-H	0.99396418	0.01136457	1.01053873	0.00779021
S61N-H	1.09445106	0.03329125	1.01190513	0.01330403
Y63N-H	1.0017121	0.00797736	0.9797166	0.0028105

L64N-H	0.87070197	0.01382415	1.03175486	0.00539705
Y67N-H	0.99553864	0.01832304	1.00089235	0.00536957
T68N-H	1.04933207	0.01547978	0.95011124	0.0047314
E69N-H	1.04075638	0.00975349	1.06698869	0.00493759
F70N-H	1.0572501	0.01609813	0.99119621	0.00512505
T71N-H	0.9962281	0.01199963	0.98448872	0.00529041
T73N-H	1.05674044	0.01230367	0.98211667	0.00506156
E74N-H	1.00372169	0.01027583	0.96730669	0.00653447
K75N-H	1.00085289	0.00770692	0.98322943	0.00452262
D76N-H	0.99175597	0.00880916	0.977907	0.0043592
E77N-H	0.9226706	0.01307327	1.00648304	0.00511562
Y78N-H	1.03965191	0.01430032	0.97290827	0.00538725
A79N-H	0.98241199	0.0169835	0.99422458	0.00490881
C80N-H	0.90221459	0.01440622	0.97337829	0.0055182
R81N-H	0.96045866	0.01599513	1.03008232	0.00574111
V82N-H	0.89184194	0.01745209	1.03911505	0.00759166
N83N-H	0.96161303	0.01413362	1.01493546	0.00584137
H84N-H	1.05471413	0.01426688	1.03803297	0.005093
L87N-H	1.01080433	0.01288621	0.98425418	0.0046213
Q89N-H	0.99059675	0.00746782	1.00754652	0.0038667
K91N-H	0.99403152	0.01380327	0.97771945	0.00504561
I92N-H	0.92330517	0.01160042	1.05965213	0.00575693
V93N-H	1.06425444	0.01580366	1.03104231	0.00594943
K94N-H	0.91941167	0.01080177	0.93125466	0.00511497
W95N-H	1.02692311	0.01480577	0.96561957	0.0056424
D96N-H	1.01380828	0.01233388	0.9714671	0.00540019
R97N-H	1.08727294	0.0108796	1.00858814	0.00491575
D98N-H	1.05231292	0.00802405	1.00954945	0.00375531
M99N-H	1.02633842	0.00658382	0.98933812	0.00297159

Table S2: A_N values obtained from ^{15}N-^{1}H HSQC spectra of 8 μM β2m + 60 nM AuNPs in the presence of 0.8 mM Tempol, with relaxation delay of 0.5 s and 5 s with corresponding errors calculated using Eq. 3 of main text.

Residue	AN_i_0.5s	Error_0.5s	AN_i_5s	Error5s
Q2N-H	1.33651231	0.10148397	1.00903533	0.04536702
R3N-H	1.20589675	0.06519262	0.97034968	0.03031392
K6N-H	1.06883175	0.05180481	1.07051463	0.02276814
V9N-H	0.93890019	0.01621791	1.06502887	0.00924142
Y10N-H	0.91811416	0.02124176	1.05252042	0.00865709
S11N-H	1.03395663	0.02713446	1.04315748	0.01049205
R12N-H	1.02772837	0.02368587	0.99669484	0.00935117
H13N-H	0.90948226	0.01888823	0.91780879	0.00850966
E16N-H	0.96223823	0.01631427	0.98076871	0.01020168
N17N-H	1.07255662	0.01679961	1.02276896	0.00915793
G18N-H	0.9619302	0.02389683	0.94810378	0.01022873
K19N-H	1.01831046	0.01653044	0.96283925	0.00729716
F22N-H	1.02510322	0.03390558	0.96892981	0.01123203
C25N-H	1.00502227	0.03082483	0.9897346	0.00940722
Y26N-H	0.97353466	0.02858267	0.96882655	0.01113233
S28N-H	1.12413699	0.05627762	1.00649414	0.02176779
G29N-H	0.94087251	0.2709228	0.8905151	0.12616055
F30N-H	0.94230906	0.03727829	1.08020308	0.01449672
S33N-H	0.79673894	0.01973138	0.90495873	0.00733088
D34N-H	0.99287341	0.01921644	1.08305488	0.01128279
I35N-H	0.99182823	0.01856906	1.09551107	0.00895083
D38N-H	0.55059316	0.009729	1.17891438	0.01111756
L39N-H	0.94898448	0.0247271	1.04417695	0.01155671
L40N-H	1.06095337	0.02891488	0.96530716	0.00934342
K41N-H	0.88102381	0.02138012	1.09895064	0.01029387
G43N-H	0.83272746	0.01815533	1.00503136	0.00999667
E44N-H	1.03260677	0.01827698	0.98287884	0.0068746
R45N-H	0.93787571	0.012146	0.94143937	0.00796416
E47N-H	1.03629434	0.01492688	1.10110942	0.00919689
K48N-H	0.99661074	0.01222786	0.96978768	0.00735651
V49N-H	1.05834825	0.0157692	0.94288076	0.0070622
S52N-H	1.02907586	0.02525333	0.98798281	0.01022339
L54N-H	1.05615403	0.01658597	0.97460657	0.00986577
S55N-H	1.12641169	0.05902998	1.05337471	0.03268205
F56N-H	0.83040973	0.02956264	1.09387146	0.02782273
D59N-H	0.98956182	0.02617021	1.04269451	0.02231889
S61N-H	0.86353631	0.05891966	0.91661396	0.02183572
Y63N-H	0.94057693	0.01218098	1.03402184	0.0061934

L64N-H	1.02439954	0.03209891	1.14466889	0.01399939
Y67N-H	1.02002073	0.02740365	0.90692607	0.00832463
T68N-H	1.05984437	0.02529573	0.90495873	0.00733088
E69N-H	0.97900596	0.01376175	1.05701033	0.00800871
F70N-H	1.05040339	0.02358583	0.987489	0.00832433
T71N-H	0.99150668	0.01772943	0.95579245	0.0084786
T73N-H	0.97630803	0.01745766	0.93018293	0.00790447
E74N-H	1.0114888	0.01446717	1.00530342	0.01025659
K75N-H	1.00845052	0.01131637	0.97286785	0.00720349
D76N-H	0.95454731	0.0130438	1.00020279	0.00746122
E77N-H	1.01343343	0.02053965	0.99065538	0.0081874
Y78N-H	1.01403517	0.02130794	0.98652187	0.00893896
A79N-H	1.01982652	0.02683031	0.88989703	0.00777924
C80N-H	1.02632336	0.02465306	0.98360791	0.00910946
R81N-H	0.96433037	0.02498724	0.96412839	0.00904532
V82N-H	1.01437281	0.03178473	0.87807653	0.00988726
N83N-H	1.03509447	0.02375699	0.94625447	0.00942608
H84N-H	1.05451988	0.02159524	1.09886443	0.00899011
L87N-H	1.07383285	0.02042347	1.067986	0.00842872
Q89N-H	0.99567552	0.01110429	0.98574578	0.00631907
K91N-H	1.00160561	0.01964228	0.95679388	0.00830286
I92N-H	0.94970099	0.01714495	1.06890695	0.00934697
V93N-H	0.95832242	0.02035441	1.01945637	0.00968115
K94N-H	1.09297263	0.01941374	1.0951915	0.00937539
W95N-H	1.09169758	0.02337923	1.01453082	0.00977338
D96N-H	1.08843282	0.0220673	0.97015598	0.00911234
R97N-H	1.02568503	0.01665015	0.9968296	0.00842127
D98N-H	1.08109976	0.01331618	0.90597994	0.00550176
M99N-H	0.98922979	0.00928562	0.98005312	0.00484405

Table S3: Signal-to-noise ratios of ^{15}N-^{1}H HSQC spectra reported in Figure S5, namely
8 μM β2m obtained with relaxation delay of 0.5 s (Spectrum A) and 5 s (Spectrum B);
8 μM β2m + 60 nM AuNPs obtained with relaxation delay of 0.5 s (Spectrum C) and 5 s (Spectrum D);
8 μM β2m + 0.8mM Tempol obtained with relaxation delay of 0.5 s (Spectrum E) and 5 s (Spectrum F);
8 μM β2m + 60 nM AuNPs + 0.8mM Tempol obtained with relaxation delay of 0.5 s (Spectrum G) and 5 s (Spectrum H).

Residue	Spectrum A	Spectrum B	Spectrum C	Spectrum D	Spectrum E	Spectrum F	Spectrum G	Spectrum H
Q2N-H	125	217	39	26	126	181	14	43
R3N-H	103	218	49	37	93	184	20	64
K6N-H	73	202	47	56	63	176	23	87
V9N-H	135	307	120	138	118	247	67	216
Y10N-H	93	308	88	145	83	262	50	231
S11N-H	95	271	84	118	85	223	43	189
R12N-H	95	278	96	125	89	221	49	210
H13N-H	102	292	97	124	95	256	56	226
E16N-H	129	241	124	112	113	199	68	192
N17N-H	156	243	147	132	136	239	72	216
G18N-H	95	245	85	107	87	205	46	190
K19N-H	157	337	137	154	131	283	70	268
F22N-H	76	255	67	100	67	211	34	174
C25N-H	74	279	70	123	58	233	37	209
Y26N-H	65	260	71	101	67	208	39	175
S28N-H	58	172	48	54	57	141	22	90
G29N-H	23	69	7	8	16	56	4	15
F30N-H	65	273	52	89	65	224	29	138
S33N-H	135	259	76	142	117	208	48	261
D34N-H	137	377	111	115	137	307	59	178
I35N-H	128	359	115	148	115	264	61	225
D38N-H	83	272	94	132	83	231	72	182
L39N-H	94	251	80	107	87	202	44	172
L40N-H	88	277	84	120	76	230	41	209
K41N-H	88	278	82	129	74	226	48	195
G43N-H	113	262	89	118	97	213	54	197
E44N-H	136	369	126	168	121	302	64	287
R45N-H	168	307	162	137	148	244	90	243
E47N-H	163	324	156	145	132	237	79	219
K48N-H	201	358	179	154	169	293	94	267
V49N-H	171	370	153	155	152	313	76	276
S52N-H	108	284	90	113	94	225	46	191
L54N-H	167	278	145	115	134	212	72	198
S55N-H	88	214	46	38	87	169	21	61
F56N-H	111	248	54	47	105	197	33	72
D59N-H	133	206	81	55	118	168	43	89

S61N-H	53	121	29	48	42	98	17	87
Y63N-H	193	558	162	200	171	467	90	324
L64N-H	89	310	70	100	90	246	36	144
Y67N-H	82	296	82	125	73	243	42	230
T68N-H	106	311	95	142	89	267	47	261
E69N-H	167	359	153	158	142	275	82	250
F70N-H	103	306	101	139	86	253	50	236
T71N-H	126	293	120	131	112	244	64	229
T73N-H	135	305	120	136	113	255	64	244
E74N-H	149	230	154	115	132	195	80	192
K75N-H	200	343	198	158	177	286	103	273
D76N-H	172	353	154	158	153	296	85	265
E77N-H	103	314	108	142	98	256	56	240
Y78N-H	113	282	104	129	96	238	54	220
A79N-H	87	321	83	131	78	265	43	244
C80N-H	90	276	92	126	88	232	47	216
R81N-H	89	290	82	124	82	231	44	215
V82N-H	73	222	70	101	72	175	36	191
N83N-H	101	278	98	116	93	225	49	206
H84N-H	116	332	110	148	97	262	55	224
L87N-H	120	336	121	152	105	280	59	238
Q89N-H	203	419	197	184	182	341	104	313
K91N-H	109	304	111	134	97	255	58	234
I92N-H	116	304	115	137	111	234	64	214
V93N-H	106	280	99	124	88	223	54	205
K94N-H	124	278	132	141	119	244	63	215
W95N-H	107	266	110	122	92	226	52	203
D96N-H	126	281	116	124	110	237	55	214
R97N-H	161	328	137	139	130	267	70	234
D98N-H	208	433	191	191	175	352	92	351
M99N-H	246	535	236	241	212	443	125	413
